# Supplementary material for: Identification of photoperiod-induced specific miRNAs in the adrenal glands of Sunite sheep (Ovis aries)
Source: Front Vet Sci. 2022 Jul 22;9:888207. doi: 10.3389/fvets.2022.888207 (PMC9354845; doi:10.3389/fvets.2022.888207)
Supplement: Supplementary file 1 [file Data_Sheet_1.ZIP › Supplementary file/miRDeep2 file.zip!/miRDeep2 file/Know_Structure/SPLP42A2/oar-mir-103.pdf]

miRBase precursor : oar-mir-103
Total read count : 16715
oar-miR-103 read count : 16714
remaining reads : 1

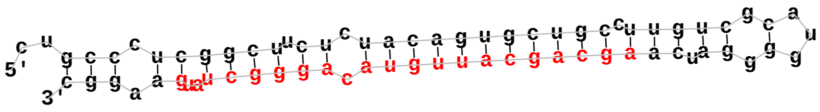

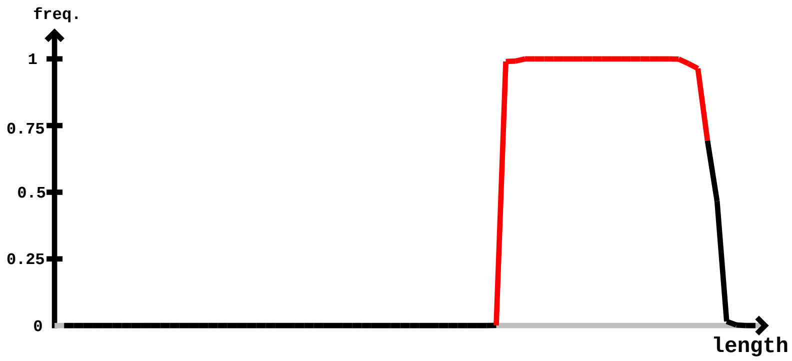


```
                                              oar-miR-103
5'- cugcccucggcuucucuacagugcugccuugucgcauggggaucaagcagcauuguacagggcuaugaaggc  -3'   exp
    ..(((.((((((.((.(((((((((((.(((((.(...).)).))))))))))))))).))))))..)).)))   reads      mm         sample
    ........ggcuucucuacagugcugccuu..........................................   1          0          seq
    ............................................aagcagcauuguacagggcuau......   1          0          seq
    ............................................aagcagcauuguacagggcuaug.....   4          0          seq
    ............................................aagcagcauuguacagggcuauga....   6          0          seq
    .............................................agcagcauuguacaggg..........   1          0          seq
    .............................................agcagcauuguacagggc.........   12         0          seq
    .............................................agcagcauuguacagggcu........   274        0          seq
    .............................................agcagcauuguacUgggcu........   1          1          seq
    .............................................agcagcauuguacagggcua.......   313        0          seq
    .............................................agcagcauuguacagggcuau......   4508       0          seq
    .............................................agcagcauuguacagggcuaug.....   3761       0          seq
    .............................................agcagcauuguacagggcuauga....   7424       0          seq
    .............................................agcagcauuguacagggcuaugaa...   215        0          seq
    .............................................agcagcauuguacagggcuaugaag..   4          0          seq
    .............................................agcagcauuguacagggcuaugaaA..   26         1          seq
    .............................................agcagcauuguacagggcuaugaaAg.   2          1          seq
    ..............................................gcagcauuguacagggcuau......   8          0          seq
    ..............................................gcagcauuguacagggcuaug.....   5          0          seq
    ..............................................gcagcauuguacagggcuauga....   11         0          seq
    ...............................................cagcauuguacagggcu........   1          0          seq
    ...............................................cagcauuguacagggcuau......   3          0          seq
    ...............................................cagcauuguacagggcuaug.....   21         0          seq
    ...............................................cagcauuguacagggcuauga....   101        0          seq
    ...............................................cagcauuguacagggcuaugaa...   8          0          seq
    ...............................................cagcauuguacagggcuaugaaA..   3          1          seq
    ...............................................cagcauuguacagggcuaugaag..   1          0          seq
```
